# Dual-emissive Polymer Dots for Rapid Detection of Fluoride in Pure Water and Biological Systems with Improved Reliability and Accuracy

**DOI:** 10.1038/srep16420

**Published:** 2015-11-10

**Authors:** Qiang Zhao, Chuanqi Zhang, Shujuan Liu, Yahong Liu, Kenneth Yin Zhang, Xiaobo Zhou, Jiayang Jiang, Wenjuan Xu, Tianshe Yang, Wei Huang

**Affiliations:** 1 Key Laboratory for Organic Electronics and Information Displays & Institute of Advanced Materials (IAM), Jiangsu National Synergetic Innovation Center for Advanced Materials (SICAM), Nanjing University of Posts & Telecommunications, 9 Wenyuan Road, Nanjing 210023, China; 2 Key Laboratory of Flexible Electronics (KLOFE) & Institute of Advanced Materials (IAM), Jiangsu National Synergetic Innovation Center for Advanced Materials (SICAM), Nanjing Tech University (NanjingTech), 30 South Puzhu Road, Nanjing 211816, China

## Abstract

It is of paramount importance to develop new probes that can selectively, sensitively, accurately and rapidly detect fluoride in aqueous media and biological systems, because F^-^ is found to be closely related to many health and environmental concerns. Herein, a dual-emissive conjugated polyelectrolyte **P1** containing phosphorescent iridium(III) complex was designed and synthesized, which can form ultrasmall polymer dots (Pdots) in aqueous media. The F^-^-responsive *tert*-butyldiphenylsilyl moiety was introduced into iridium(III) complex as the signaling unit for sensing F^−^ with the quenched phosphorescence. Thus, the dual-emissive Pdots can rapidly and accurately detect F^−^ in aqueous media and live cells as a ratiometric probe by measuring the change in the ratio of the F^−^-sensitive red phosphorescence from iridium(III) complex to the F^−^-insensitive blue fluorescence from polyfluorene. Moreover, the interaction of Pdots with F^−^ also changes its emission lifetime, and the lifetime-based detection of F^−^ in live cells has been realized through photoluminescence lifetime imaging microscopy for the first time. Both the ratiometric luminescence and lifetime imaging have been demonstrated to be resistant to external influences, such as the probe’s concentration and excitation power. This study provides a new perspective for the design of promising Pdots-based probes for biological applications.

As one of the important inorganic anions, fluoride anion (F^−^) plays a great role in many health and environmental concerns. In particular, reasonable water fluoridation and addition of fluoride to toothpaste have become a widespread practice due to the beneficial effects of fluoride on dental health and osteoporosis treatment^1^,^2^,^3^,^4^. However, excessive fluoride intake may trigger adverse effects^5^. It can not only result in dental or skeletal fluorosis but also be associated with kidney failure and nephrolithiasis. The EPA (United States Environmental Protection Agency) has set a maximum contaminant level of 4 mg L^−1^ (4 ppm or 211 μM) in drinking water^6^. Thus it is of great importance to develop effective strategy that can selectively, sensitively, accurately and rapidly detect fluoride anion in aqueous media and biological systems.

Currently, two standard fluoride-detecting methodologies have been recommended by WHO, including ion-selective electrode and ion chromatography. Both the techniques, however, require professional equipments and well-trained staff, and are difficult to be performed in those poor or even middle-income sufferers. Hence, a convenient and selective detecting methodology is highly desirable for the practical purpose. Fluorescent probes for F^−^, with features of non-invasive sensing, real-time detection, easy operation, high sensitivity and selectivity, have received increasing interest^7^,^8^,^9^,^10^,^11^,^12^,^13^,^14^,^15^. Because of the extraordinary electronegativity and small size of fluorine atom, most of current F^−^ sensing mechanisms focus on the hydrogen bonding, anion-π and Lewis acid/base interactions^16^,^17^,^18^,^19^,^20^,^21^. However, most of them can only detect tetrabutylammonium fluoride in organic solvents rather than inorganic fluoride salts in aqueous media. And the fluoride sensing or signaling has been proved to be very difficult in aqueous solution due to the high hydration enthalpy (∆*H*o = −504 kJ/mol) of fluoride^7^. Most recently, a well-established strategy for detecting F^−^ in aqueous media is based on the unique chemical reactivity between silicon and fluoride^22^,^23^,^24^,^25^,^26^,^27^. Compared to the sensors based on noncovalent and weak interaction, these specific reaction-based probes exhibit higher selectivity and stability^20^. In spite of these merits, most of the reported probes are water-insoluble and usually suffer from long response time to ensure complete reaction, limiting their applications in real-time detection. In addition, most probes are turn-on or turn-off with the change in single-wavelength-intensity as the reporting signal, and the accuracy of detection is easily influenced by measurement conditions and environment, such as the probe’s concentration, excitation power, and autofluorescence interference.

To solve the above problems, we hope to develop ratiometric luminescence and lifetime-based probes. Ratiometric luminescence detection, measuring the ratio changes of the emission intensity at two different wavelengths, allows more accurate F^−^ detection especially in intracellular and *in vivo* detection, because they are endowed with a built-in correction for environmental effects^28^,^29^,^30^,^31^. In addition, lifetime-based detection, which is independent of the probe concentration, the power of laser source and photobleaching, is another powerful method to greatly improve the sensing sensitivity and reliability by utilizing the changes in emission lifetime of a probe upon interaction with the analyte^32^,^33^,^34^,^35^,^36^. Especially, the lifetime-based detection and imaging of intracellular analytes become feasible with the rapid development of photoluminescence lifetime imaging microscopy (PLIM).

Herein, we introduced F^−^-responsive *tert*-butyldiphenylsilyl (TBDPS) moiety into the ligand of red-emitting phosphorescent iridium(III) complex (**1**, Fig. 1), which was covalently bonded to the mainchain of blue-emitting polyfluorene-based conjugated polyelectrolyte (CPE) (**P1**, Fig. 1). CPE is a kind of excellent bioprobe because of its enhanced sensing sensitivity due to the “molecular wires” effect, high light absorptivity and fluorescence brightness, excellent photostability and fine biocompatibility^37^,^38^,^39^,^40^,^41^,^42^. In addition, due to their amphiphilic structure of hydrophobic backbones and hydrophilic side chains, CPE could self-assemble to form ultrasmall nanoparticles with the size of less than 20 nm in aqueous solution without any further modifications and other auxiliary components, which are called as polymer dots (Pdots). All of these characteristics are advantageous for their applications in biosensing and bioimaging. A phosphorescent iridium(III) complex was selected as the signaling unit due to its excellent photophysical properties, especially long emission lifetime, which is very favorable for lifetime-based detection^43^,^44^,^45^,^46^,^47^,^48^,^49^. By measuring the change in the ratio of the F^−^-sensitive red phosphorescence from iridium(III) complex to the F^−^-insensitive blue fluorescence from polyfluorene, the dual-emissive Pdots can rapidly and accurately detect F^−^ in aqueous media and live cells as a ratiometric probe. Furthermore, the lifetime-based detection of F^−^ in live cells has been realized through PLIM for the first time, and the advantages of lifetime detection have been demonstrated successfully.

## Results

### Synthesis and characterization of 1 and P1

The synthetic procedue of model complex **1** was shown in Fig. S1. For the synthesis of polymer **P1**, two routes have been adopted (Fig. 2), namely the coordination-polymerization method (route I) and the polymerization-coordination method (route II). First, route I was tried to synthesize polymer **P1**. The iridium(III) complex monomer **1’** was synthesized, which was then copolymerized with fluorene-based monomers by Suzuki coupling reaction. However, almost no target polymer **P1** was generated, maybe due to the steric hindrance of two bulky TBDPS moieties in the ligands of **1’**, which made the Suzuki coupling reaction difficult to take place. Then, route II was carried out. The macromolecular ligand **Pr1** was synthesized first through Suzuki coupling reaction, which then reacted with cyclometalated iridium(III) chloro-bridged dimer. Thus, the target polymer **P1** was obtained successfully. The detailed synthetic procedue of **P1** was shown in Scheme S2. The chemical structures of **1** and **P1** were characterized by ^1^H and ^13^C NMR. The weight-average molecular weight of **P1** was 6400 with polydispersity index of 1.20 as determined by gel permeation chromatography. The actual content of iridium(III) complex in **P1** was estimated to be 11.4 mol% via ^1^H NMR. Due to its amphiphilic structure, **P1** can form the well-dispersed and ultrasmall Pdots in the phosphate buffer solution (PBS) with a diameter of <10 nm from transmission electron microscopy (TEM) via self-assembly (Fig. 3a). The hydrodynamic diameter of the Pdots was determined by dynamic light scattering (DLS), the mean diameter of the Pdots is about 16 nm (Fig. 3b). It is reasonable that hydrodynamic diameter of the Pdots measured by DLS is larger than their size obtained by TEM, because the hydrodynamic diameter is the hydrated diameter combined by the Pdots cores together with the solvent coating layer, while for TEM, this hydration layer is not present^50^.

### Optical response of 1 to F^−^

**Figure 1:**
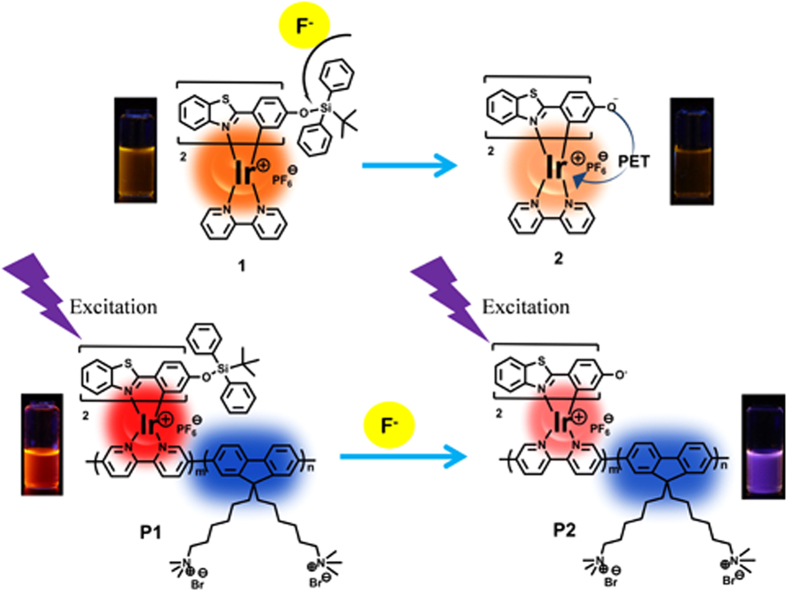
Chemical structures of the model complex 1 (a) and polymer P1 (b) and their sensing mechanism.

**Figure 2:**
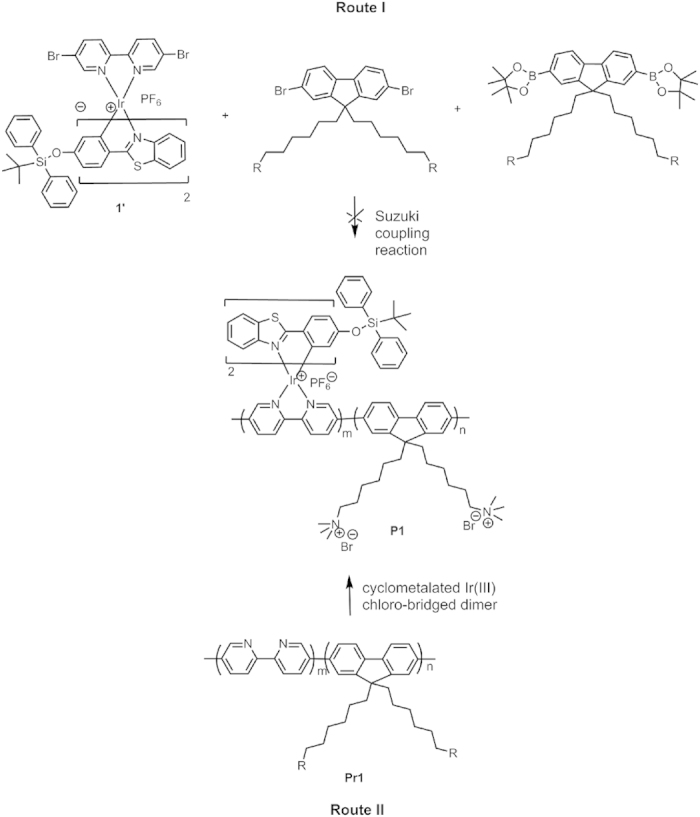
Synthetic routes of the target conjugated polyelectrolyte P1.

**Figure 3:**
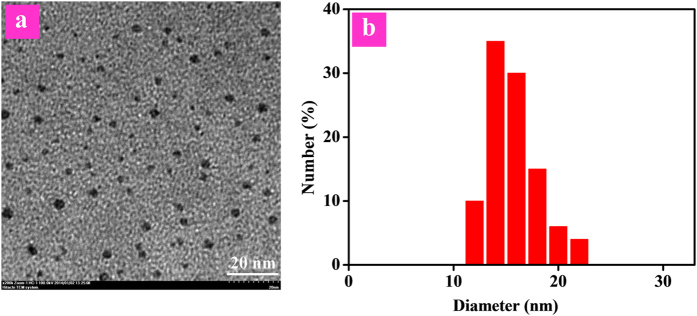
TEM image and DLS measurement of P1 in aqueous solution.

**Figure 4:**
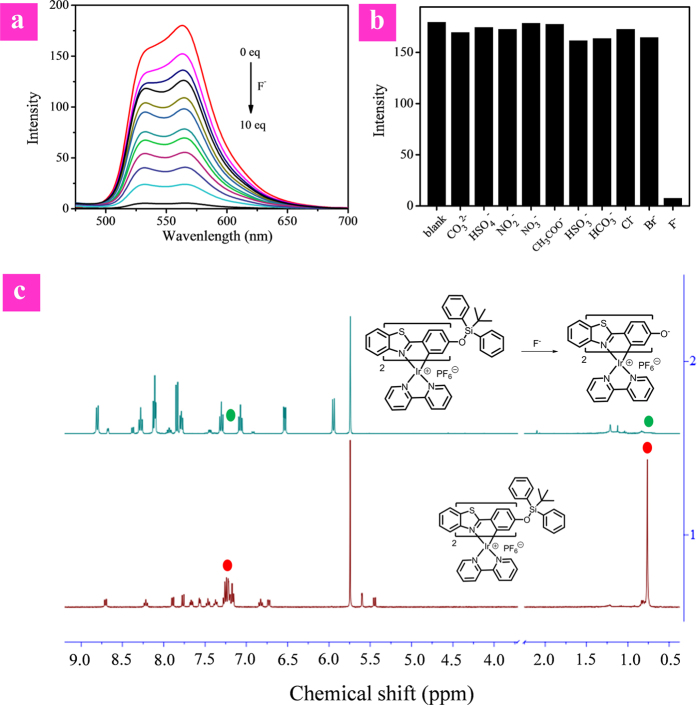
(**a**) PL spectra of **1** in THF (20 μM) with addition of F^−^ upon excitation at 365 nm. (**b**) Luminescent intensity of **1** in THF upon the addition of various anions (40 eq.). (**c**) ^1^H NMR spectral changes for **1** before and after addition of F^−^.

To evaluate the feasibility of our design strategy, the F^−^-sensing performance of model complex **1** was investigated in tetrahydrofuran solution with tetrabutylammonium fluoride as F^−^ source because of the hydrophobicity of **1**. As shown in Fig. 4a, complex **1** exhibits two emission bands at 533 nm and 563 nm. The emission intensity of **1** decreased evidently and progressively with the addition of F^−^ from 0 to 10 equivalents. The response of **1** to F^−^ was very fast, which could be finished within one minute (Fig. S5). To further investigate the interaction between complex **1** and F^−^, ^1^H NMR spectra of complex **1** were recorded in the presence and absence of F^−^ (Fig. 4c). Before F^−^ addition, the characteristic signals (~0.76 ppm and ~7.25 ppm) expected from the *tert*-butyl and two phenyl groups of the TBDPS moiety were visible. These signals disappeared in the presence of 5.0 equiv of F^−^. The results suggest that in the presence of F^−^, the Si-O bond-breaking of complex **1** takes place to afford complex **2** (Fig. 1). This process has been further demonstrated by MALDI-TOF of complex **1** before and after addition of F^−^ (Fig. S4). In order to further confirm the mechanism, complex **2** was synthesized and characterized by MALDI-TOF and ^1^H NMR, which are same to those of the reaction product between complex **1** and F^−^, verifying the sensing mechanism as shown in Fig. 1a. The quenched phosphorescence of the product **2** may be attributed to the photoinduced electron transfer (PET) from the electron-rich oxygen atom to the excited metal complex.

For further evaluation of the fluoride sensing ability of **1**, the selectivity was tested by addition of 40 equivalents of common anions, including Cl^−^, Br^−^, CH_3_COO^−^, NO_3_^−^, NO_2_^−^, HSO_4_^−^, HCO_3_^2−^, CO_3_^2−^, HSO_3_^−^ and F^−^. As shown in Fig. 4b, only F^−^ induced the decrease of emission intensity and all other anions did not cause evident changes of the emission spectra. This result indicated that **1** is a promising probe for recognizing fluoride ions over other common anions.

### Luminescence response of P1 to F^−^

**Figure 5:**
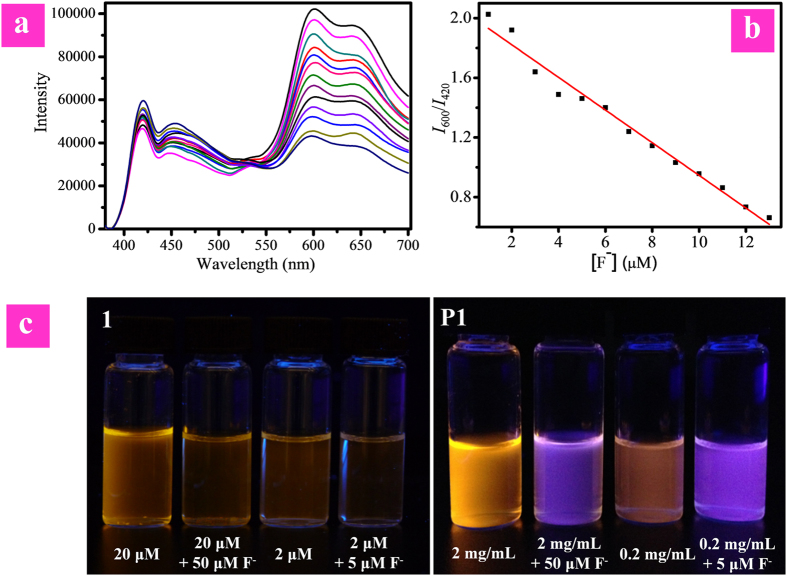
(**a**) PL spectra of **P1** in PBS (pH = 7.4) with addition of F^−^ from 0 to 13 μM upon excitation at 365 nm. (**b**) *I*_600_/*I*_420_ as a function of [F^−^] and its trendline. (**c**) Photographs of complex **1** in CH_3_CN and polymer **P1** in PBS solution under excitation at 365 nm.

The sensitive response of complex **1** to F^−^ endowed **P1** with excellent sensing properties by introducing it into the hydrophilic CPE mainchain. Using the F^−^-insensitive blue fluorescence from polyfluorene as the reference signal, **P1** fulfilled the features of ratiometric probe for detecting F^−^ in aqueous media. The F^−^ sensing peformance of **P1** was investigated in PBS (pH 7.4 at 25 °C) using NaF as F^−^ source. The concentration of **P1** was calculated based on the repeating units. As shown in Fig. 5a, in the absence of fluoride ions, the intensity of blue fluorescence with maximum peak at 420 nm from polyfluorene was moderate, and that of red phosphorescence at about 600 nm from iridium(III) complex was stronger. At first, the quantum yields of the probe is 0.09. Upon addition of F^−^, the intensity of red emission band decreased gradually, while that of blue emission is almost unchanged. The difference between two emission wavelengths was large (180 nm) and there was almost no overlap between the two emission bands, which led to accurate measurement of two emission intensities to yield the ratiometric value. While the F^−^ concentration achieved 13 μM, the quantum yields value became 0.03. As shown in Fig. 5b, the ratios of emission intensity at 600 nm to that at 420 nm (*I*_600_/*I*_420_) exhibited a dramatic change from 2.03 to 0.66 (Fig. 5b). *I*_600_/*I*_420_ as a function of F^−^ concentration exhibits a good linear relationship within the F^−^ concentration from 5 to 13 μM. As the therapeutic dosing level is below 52.6 μM, our probe **P1** is promising in future clinical applications. The detection limit of **P1** was determined to be 7.0 ppb, which was significantly lower than the suggested maximum limit of fluoride anion in drinking water, showing good sensitivity. It was fascinating that the response of **P1** to F^−^ is very fast, and the reaction between **P1** and F^−^ can be finished within two minutes. These results clearly indicated that **P1** is an excellent ratiometric F^−^ probe. In addition, the large ratiometric value allowed distinct visualization of the change in emission color of solution under UV lamp. In the absence of F^−^, the emission color of solution was orange. It turned to blue after addition of F^−^. This evident change of emission color enabled nake-eye detection of F^−^ (Fig. 5c).

The resistance of sensing performance of **1** and **P1** to probe concentration was illustrated in Fig. 5c. The emission intensity of complex **1** decreased evidently upon reducing its concentration from 20 μM to 2 μM (Fig. 5c), which was similar to the result upon reaction with F^−^. Thus, it was difficult to distinguish whether the quenching of emission intensity is due to the reaction of complex **1** with F^−^ or just at low concentration of complex **1**. Additionally, the change in emission color of complex **1** at 2 μM in the absence and presence of F^−^ was not as remarkable as that observed for **1** at 20 μM, indicating that concentration made a big difference to the sensing performance of single-wavelength-intensity based probes. In contrast, both the solutions of **P1** at the concentration of 2.0 mg/mL and 0.2 mg/mL exhibited orange light under UV lamp, and their emission colors changed to blue upon reaction with F^−^, indicating that the change in emission color is induced by F^−^ instead of concentration. Hence, the concentration of **P1** has little impact on the sensing performance due to the ratio change of the emission intensity at two different wavelengths, demonstrating that ratiometric probe **P1** was able to fulfill accurate detection of F^−^.

Furthermore, to investigate the pH range of probe worked, the F^−^ titration experiments of **P1** under different pH condition (9.2 or 4.0) have also been carried out. As shown in Figure S10, **P1** exhibits excellent sensing performance under both pH values. Hence, we think that polymer **P1** can work in the pH range of 4.0~9.0.

### Ratiometric imaging of intracellular F^−^

**Figure 6:**
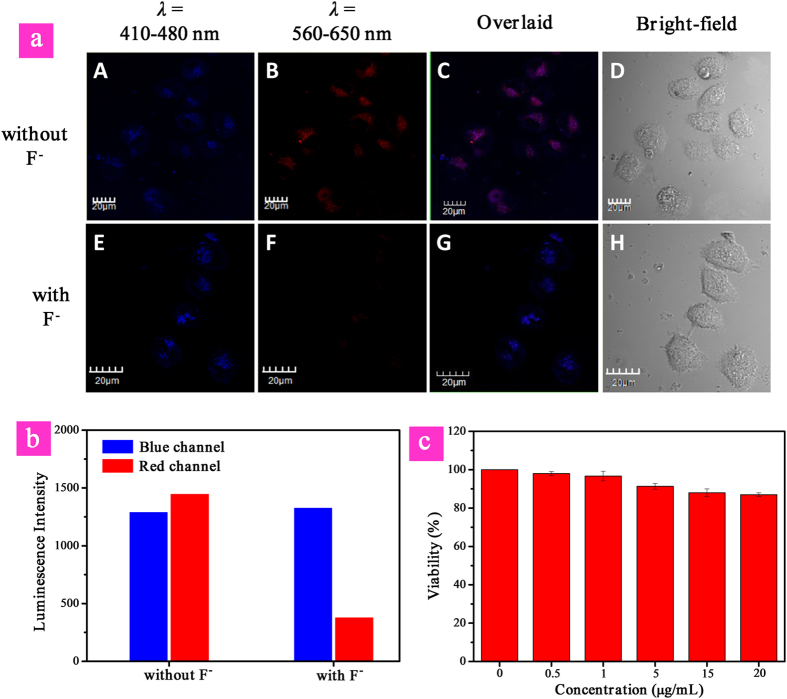
(**a**) Confocal microscopy images of HeLa cells treated with **P1** (top), followed by incubation with NaF (bottom) (*λ*_ex_ = 405 nm) of HeLa cells labeled with **P1** incubated with and without F^−^ observed at emission wavelengths of 410–480 nm and 560–650 nm. (**b**) Luminescence intensity of HeLa cells recorded at emission wavelengths of 410–480 nm (blue channel) and 560–650 nm (red channel). (**c**) MTT cell viability values (%) of HeLa cells incubated with **P1** at various concentrations for 24 h. Scale bar is 20 μM.

To realize F^−^ sensing in biological systems, such as live cells, the cytotoxicity of **P1** was evaluated by the MTT [3-(4,5-dimethylthiazol-2-yl)-2,5-diphenyltetrazolium bromide] assay. As shown in Fig. 6c, the high cell viability (>85%) indicates that **P1** has excellent biocompatibility and low cytotoxicity. Therefore, **P1** is preferable for live cell imaging and intracellular F^−^ detection. Next, the intracellular F^−^ sensing performance of **P1** was investigated. We first incubated the cells with **P1** for 60 min at 37 °C. After cellular uptake, we washed the cells with PBS buffer to remove the free **P1** in solution and on the cell surface. Then the experimental group was further incubated with F^−^ (20 μM) for 30 min at 37 °C, and the control group was directly incubated for 90 min. Fig. 6a shows the confocal fluorescence images of HeLa cells treated with **P1** in the presence or absence of F^−^ at 37 °C. The excitation wavelength was 405 nm. It can be seen that for the live cells before and after treated with F^−^, no evident change was observed for the images collected at 410 to 480 nm (blue channel), which corresponded to the fluorescence from polyfluorene. However, the emission intensity of images recorded at 560–650 nm (red channel), which corresponded to the phosphorescence from iridium(III) complex in **P1**, decreased evidently in the presence of F^−^ compared with that without F^−^. Thus, the intensity ratio of the red channel over the blue channel (Fig. 6b), *I*_red_/*I*_blue_, was determined to decrease from 1.12 to 0.29 upon F^−^-induced reaction of **P1**. This result showed that excellent ratiometric sensing F^−^ in live cells can be realized like that in PBS buffer.

**Figure 7:**
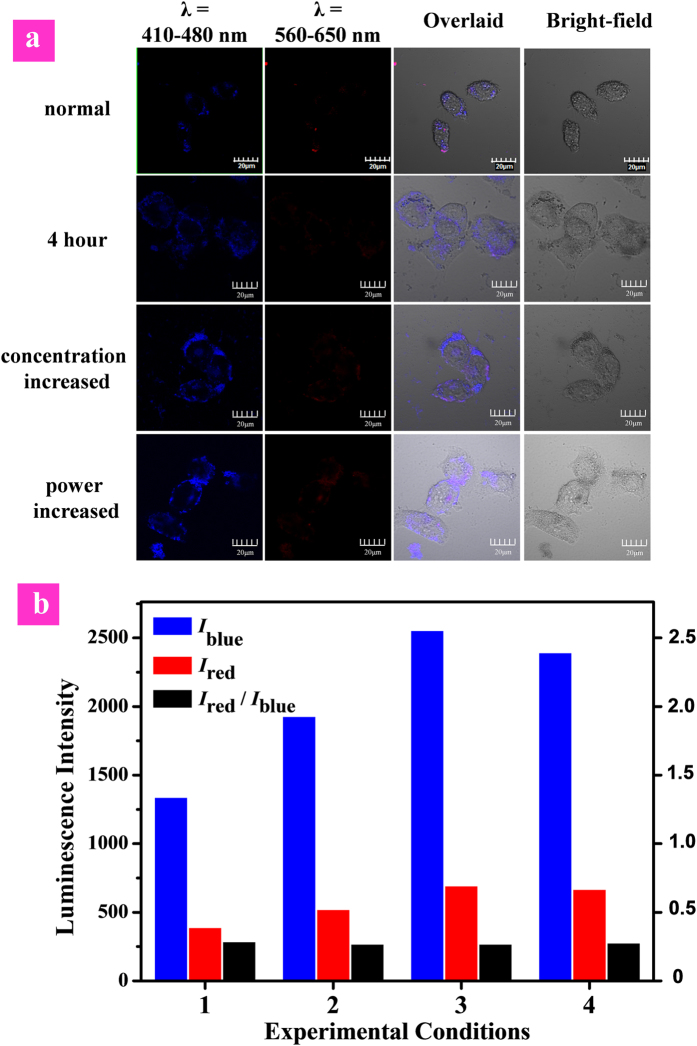
Luminescence images (a) and intensity (b) of HeLa cells treated with P1 followed by incubation with NaF at different experimental conditions: (1) normal, laser power is normal, 3 h; (2) the incubation duration was increased to 4 h; (3) the P1 concentration for incubation was increased from 1.0 × 10^–2^ mg/mL to 2.0 × 10^–2^ mg/mL; (4) the 405 nm laser power was increased. Scale bar is 20 μm.

In order to demonstrate the resistance of the ratiometric detection to the influence of external factors in live cells, we investigated the intracellular sensing under different experimental conditions as shown in Fig. 7. Both the intensity of red phosphorescence from iridium(III) complex (*I*_red_) and blue fluorescence from polyfluorene (*I*_blue_) were disturbed by the changes in experimental conditions, such as that (1) the incubation duration was increased to 4 h; (2) the dose of **P1** was increased from 1.0 × 10^−2^ mg/mL to 2.0 × 10^−2^ mg/mL; (3) the 405 nm laser power was randomly increased. Although the blue- or red-channel emission intensity (*I*_blue_ or *I*_red_) measured at different experimental conditions showed the evident change, the *I*_red_/*I*_blue_ values exhibited little fluctuation (Fig. 7b). This excellent resistance of the ratiometric detection toward external influences demonstrated that ratiometric probes provided more accurate and precise sensing results than those turn-on or turn-off probes based on single-wavelength-intensity as the reporting signal.

### Lifetime imaging of intracellular F^−^

**Figure 8:**
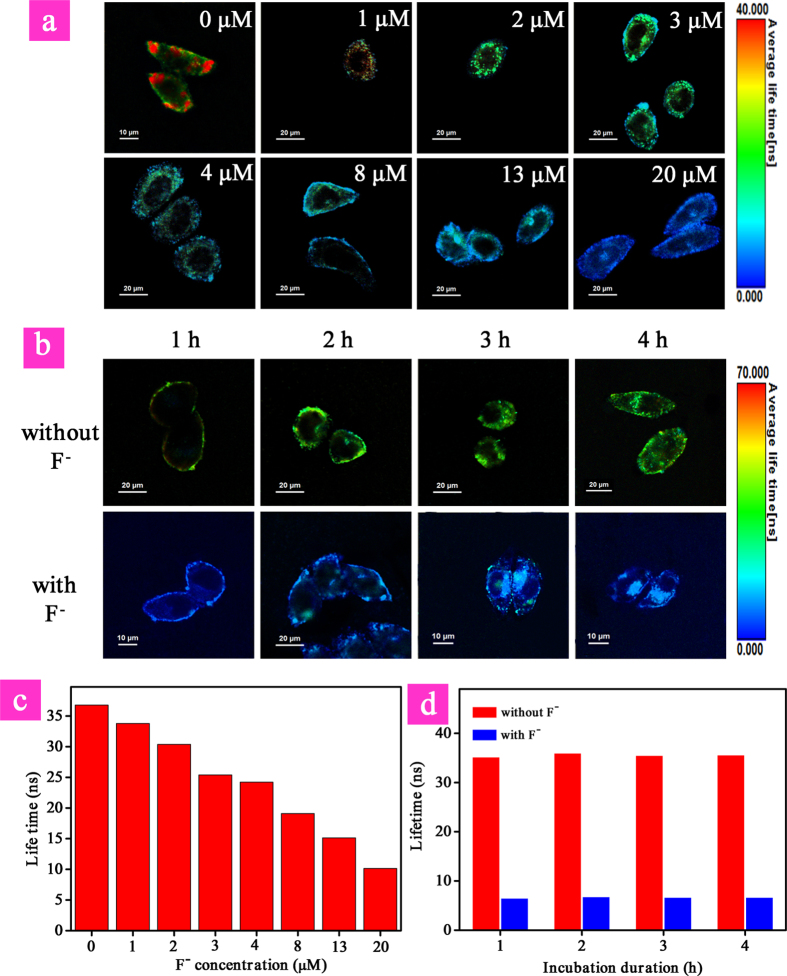
Photoluminescence lifetime imaging (a) and average lifetimes (c) of HeLa cells treated with P1 for 3 h in the presence of different concentration of F^−^. Photoluminescence lifetime imaging (**b**) and average lifetimes (**d**) of HeLa cells treated with **P1** from 1 h to 4 h in the absence and presence of 30 μM F^−^. Scale bar is 20 μM.

Taking advantage of the long emission lifetime (τ) of phosphorescence signal from iridium(III) complex, the photoluminescence lifetime imaging of different concentration of intracellular F^−^ was performed. As shown in Fig. 8a,c, significant difference in average emission lifetime was observed. In the absence of F^−^, the average emission lifetime of **P1** was about 36.0 ns, while it decreased evidently in the presence of increasing concentration of F^−^ from 1 μM to 20 μM due to the quenching of long-lived phosphorescent signal. When HeLa cells were treated with **P1** in the presence of 20 μM F^−^, the lifetime was decreased to about 6.8 ns. Hence, the monitoring of variation in intracellular F^−^ has been realized by lifetime imaging. Furthermore, resistance of the lifetime as the sensing signal toward external influences was investigated. The same lifetimes have been observed when changing the incubation duration of **P1** from 1 h to 4 h in the absence and presence of 30 μM F^−^ (Fig. 8b,d). This indicated the advantage of lifetime-based imaging which was resistant to external interferences, such as incubation time and probe concentration. Simultaneously, as a kind of time-resolved luminescence imaging technique, PLIM allows the signal of **P1** to be recognized from the interferences of short-lived background fluorescence and scattered light based on their difference in emission lifetimes, improving the reliability and stability of F^−^ detection.

## Discussion

In summary, a reaction-based fluorescent/phosphorescent dual-emissive nanoprobe **P1** has been designed and synthesized. This class of nanoprobe can solve the key issues during the F^−^ sensing process. Firstly, the hydrophilic characteristic of CPE enables the probe to be soluble in water. Secondly, the reaction-based sensing mechanism ensures the detection in aqueous media and biological systems with high selectivity and rapid response time. Thirdly, both the ratiometric luminescence and lifetime detection have been demonstrated to be resistant to external influences, effectively improving the sensing reliability and accuracy. It is noteworthy that photoluminescence lifetime imaging of intracellular F^−^ has been realized for the first time. Importantly, we believe that the design strategy for ratiometric and lifetime probes based on CPE in this work is not limited to fluoride sensing; the incorporation of responsive phosphorescent complex as reporters and insensitive polymer backbone as internal standard into the CPE will lead to new ratiometric luminescence and lifetime probes for specific analytes.

## Methods

### Materials and Methods

All chemical reagents, unless otherwise specified, were purchased from Sigma Aldrich Chemical Company. All solvents for reaction and photophysical investigation were of HPLC grade. IrCl_3_∙3H_2_O was an industrial product and used without further purification. Photoluminescence (PL) spectra were measured on a Perkin Elmer LS-55 with Xe lamp excitation source and a Hamamtsu (Japan) 928 PMT, using a 90 degree angle for solution samples. UV-vis absorption spectra were recorded on a UV-1700 Shimadzu UV-vis spectrophotometer. Nuclear magnetic resonance (NMR) spectra were recorded on Bruker ACF400 (400 MHz) instrument. Mass spectra were obtained on a Bruker autoflex matrix-assisted laser desorption ionization time-of-flight (MALDI-TOF/TOF) mass spectrometer (MS_3_) and a Shimadzu GCMS-QP2010. The gel permeation chromatography (GPC) analysis of the polymers was conducted on a Shimadzu 10 Å with THF as the eluent and poly(styrene) as standard. The data were analyzed by using the software package provided by Shimadzu Instruments. Photographs of the solution samples were taken with a Cannon EOC 400D digital camera under a hand-held UV lamp. Average particle size was measured by laser light scattering (LLS) with particle sizing software (90 plus, Brookhaven Instruments Co. USA) at a fixed angle of 90° at room temperature. Transmission electron microscopy (TEM) was conducted on a JEOL JEM-2100 Transmission electron microscopy at an acceleration voltage of 150 kV.

### Cell culture

The human cervical cancer HeLa cell line was obtained from the American Type Culture Collection and cultured in Dulbecco’s modified Eagle’s medium (Gibco BRL, Gaithersburg, MD) supplemented with 10% heat-inactivated fetal bovine serum, 2 mM glutamine, 100 U/mL penicillin, and 100 μg/mL streptomycin (Gibco BRL, Gaithersburg, MD). Cells were cultured at 37 °C in a humidified chamber containing 5% CO_2_.

### Confocal luminescence imaging

Confocal luminescence imaging was carried out on an Olympus IX81 laser scanning confocal microscope equipped with a 40 immersion objective len. A semiconductor laser at 405 nm was served as excitation of the HeLa cells incubated with Pdots nanoprobes. The HeLa cells were incubated with the Pdots (10 μg/mL) nanoprobes at 37 °C for 3 h, then incubated with and without F^−^ for another 30 min. Then the cells were immediately transferred into Live Cell Imaging System (OLYMPUS, Xcellence) for confocal luminescence imaging. The emission was collected at 410–480 nm and 560–650 nm for the HeLa cells incubated with Pdots nanoprobes.

### Photoluminescence lifetime imaging

The HeLa cells were incubated with the Pdots nanoprobe (10 μg/mL) at 37 °C for 3 h, and then with different concentration of F^−^ for another 30 min. The PLIM setup is integrated with Olympus IX81 laser scanning confocal microscope. The luminescence signals were detected by confocal microscope system and the correlative calculation of the data was performed using professional software which was provided by PicoQuant GmbH. The excitation light of 405 nm with a frequency of 0.5 MHz from the pulse diode laser (PicoQuant, PDL 800-D) was focused onto the sample with a 40 X objective lens (NA 0.95) for single-photon excitation. The luminescence signals were collected in the range of 410–650 nm.

### Synthesis of polymer Pr1

The monomer **M1** (0.032 mmol, 10.0 mg), **M2** (0.068 mmol, 44.2 mg), **M3** (0.10 mmol, 74.4 mg), [Pd(PPh_3_)_4_] (5 mg) and tetrabutylammonium bromide (TBAB) were placed in a reaction tube. A mixed solvent of toluene (3.3  mL) and K_2_CO_3_ aqueous solution (2.2 mL, 2M) were added. The reaction vessel was degassed and the reaction was stirred vigorously at 85 °C for 48 h under the protection of nitrogen-atmosphere at dark environment. Then, the reaction was cooled down to room temperature and precipitated in methanol. The polymer was filtered and washed with methanol and acetone, and then dried under vacuum at RT for 24 h to obtain the polymer Pr1 in the yield of 75%. ^1^H NMR (400 MHz, CDCl_3_, δ): 9.06 (s, 1H), 8.59 (s, 1H), 8.16 (s, 2H), 7.39–7.86 (m, 21H), 3.30 (t, 12H), 1.65 (m, 60H). ^13^C NMR (100 MHz, CDCl_3_, δ): 151.50, 140.52, 140.12, 128.85, 127.21, 126.37, 121.32, 121.00, 120.19, 83.81, 55.33, 40.30, 34.02, 33.35, 32.62, 29.71, 29.08, 27.77, 25.00, 23.72.

### Synthesis of polymer Pr2

Trimethylamine in tetrahydrofuran (1 mL, 2 mmol/L) was added dropwise to a solution of polymer **Pr1** (40 mg) in THF (8 mL) at RT. The reaction was stirred at RT for 12 h. Then, the precipitate was redissolved by methanol (6 mL). Additional trimethylamine in tetrahydrofuran (1 mL, 2 mmol/L) was added, and the reaction was stirred at RT for 24 h. After removal of solvent, acetone was added to the precipitate. The polymer was dried under vacuum for 24 h to obtain the light yellow powder with the yield of 87%. ^1^H NMR (400 MHz, d6-DMSO, δ): 9.12 (s, 1H), 8.59 (br, 3H), 7.73 (m, 21H), 3.05 (s, 60H), 2.28 (br, 24H), 0.77–1.61(m, 36H). ^13^C NMR (100 MHz, (CD_3_)_2_SO, δ): 126.52, 121.25, 72.22, 65.96, 65.59, 60.71, 52.51, 35.49, 32.43, 29.34, 28.57, 27.46, 25.97, 22.48, 15.62.

### Synthesis of polymer P1

Polymer **Pr2** and cyclometalated Ir(III) chloro-bridged dimer **L3** were added to a 150 mL flash. A mixed solvent of methanol (6 mL) and dichloromethane (6 mL) were added. The reaction vessel was degassed and the reaction was stirred vigorously at 50 °C for 8 h under the protection of nitrogen-atmosphere at dark environment. When finished, the solution was cooled down to room temperature and then a 5-fold excess of potassium hexafluorophosphate was added. The suspension was stirred for another 2 h and then was filtered to remove insoluble inorganic salts. The product was concentrated and precipitated in acetone. The polymer was dried under vacuum for 24 h to obtain the light red powder. ^1^H NMR (400 MHz, (CD_3_)_2_SO, δ): 7.45–8.48 (m, 31H), 7.37 (br, 2H), 7.18 (br, 5H), 6.89 (br, 1H), 3.06 (d, 60H), 2.19 (br, 8H), 0.76–1.48 (m, 60H). ^13^C NMR (100 MHz, CDCl_3_, δ): 136.82, 134.94, 129.65, 127.98, 52.51, 30.89, 29.47, 28.96, 27.01, 26.43, 25.95, 24.21, 22.47, 19.14.

The synthetic routes of the CPEs were shown in Scheme S2 in Supporting Information (SI).

## Additional Information

**How to cite this article**: Zhao, Q. *et al.* Dual-emissive Polymer Dots for Rapid Detection of Fluoride in Pure Water and Biological Systems with Improved Reliability and Accuracy. *Sci. Rep.* **5**, 16420; doi: 10.1038/srep16420 (2015).

## Supplementary Material

Supporting Information
