# Supplementary material for: Dual-emissive Polymer Dots for Rapid Detection of Fluoride in Pure Water and Biological Systems with Improved Reliability and Accuracy
Source: Sci Rep. 2015 Nov 10;5:16420. doi: 10.1038/srep16420 (PMC4639760; doi:10.1038/srep16420)
Supplement: Supporting Information [file srep16420-s1.pdf]

Supporting Information

Dual-emissive Polymer Dots for Rapid Detection of Fluoride in Pure Water and Biological Systems with Improved Reliability and Accuracy

Qiang Zhao[1,*], Chuanqi Zhang[1], Shujuan Liu[1], Yahong Liu[1], Kenneth Yin Zhang[1], Xiaobo Zhou[1], Jiayang Jiang[1], Wenjuan Xu[1], Tianshe Yang[1], and Wei Huang[2,*]

[1]Key Laboratory for Organic Electronics and Information Displays & Institute of Advanced Materials (IAM), Jiangsu National Synergetic Innovation Center for Advanced Materials (SICAM), Nanjing University of Posts & Telecommunications, 9 Wenyuan Road, Nanjing 210023, China.

[2]Key Laboratory of Flexible Electronics (KLOFE) & Institute of Advanced Materials (IAM), Jiangsu National Synergetic Innovation Center for Advanced Materials (SICAM), Nanjing Tech University (NanjingTech), 30 South Puzhu Road, Nanjing 211816, China.

*iamqzhao@njupt.edu.cn ; wei-huang@njtech.edu.cn

1. Synthesis

The ligands and Ir(III) complexes were synthesized according to the route depicted in Fig. S1.

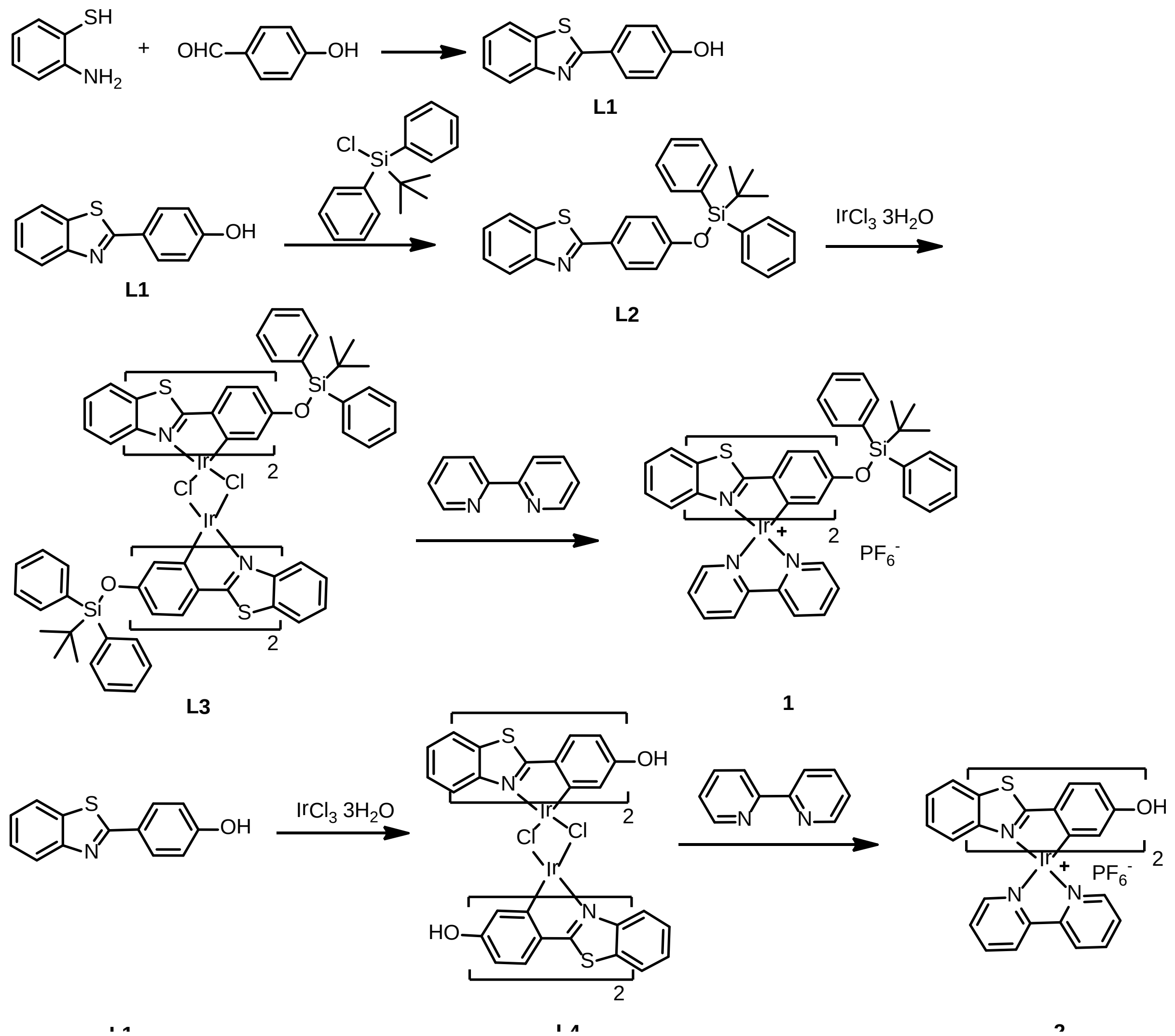

Figure S1. Synthesis of the ligands **L1** and **L2**, and their IrIII complexes **1** and **2**.

Synthesis of L1: The N,N-dimethylformamide (5.0 mL) was added to a flask containing 2-aminothiophenol (4.0 g) and 4-hydroxybenzaldehyde (4.12 mL). The mixture was refluxed for 72 h and the temperature of the reaction system was raised to 110 ^{o}C. Then, the reaction was extracted with water and ethyl acetate. The solvents were evaporated in vacuum and the product was recrystallized from ethanol. Yield: 80%. ^{1}H NMR (400 MHz, d6-DMSO) δ:

10.21 (s, 1H), 8.07 (d, J = 7.9 Hz, 1H), 8.00 - 7.87 (m, 3H), 7.53 - 7.45 (m, 1H), 7.43 - 7.33 (m, 1H), 6.97 - 6.88 (m, 2H).

Synthesis of L2: The N,N-dimethylformamide (5.0 mL) was added to a flask containing tert-butyl(chloro)- diphenylsilane (2.0 mL), imidazole (200 mg) and **L1** (1 g). The mixture was refluxed for 24 h under nitrogen. Then the reaction was extracted with water and ethyl acetate. The solvents were evaporated in vacuum and the product was recrystallized from ethanol. Yield: 80%. ^{1}H NMR (400 MHz, d6-DMSO) δ: 6.91 - 6.86 (dd, 2H), 7.51 - 7.39 (m, 8H), 1.05 (s, 9H), 7.71 - 7.66 (m, 4H), 7.96 - 7.79 (d, J = 7.8 Hz, 1H), 8.09 - 8.04 (d, 1H), 7.91 - 7.86 (m, 2H).

Synthesis of L3: A mixture of 2-ethoxyethanol and water (3:1, v/v) was added to a flask containing $IrCl_3 \cdot 3H_2O$ (1 mmol) and ligand **L2** (2.5 mmol). The mixture was refluxed for 24 h. After cooling down, the red solid precipitate was filtered and washed with ethanol to give crude cyclometalated Ir(III) chloro-bridged dimer.

Synthesis of 1: The solution of cyclometalated Ir(III) chloro-bridged dimer **L3** (200 mg) and *2,2'*-bipyridine (64 mmol) in CH2Cl2/MeOH [30 mL, 2:1 (v/v)] was heated to reflux. After 4 h, the red solution was cooled down to room temperature and then a 5-fold excess of potassium hexafluorophosphate was added. The suspension was stirred for 1 h and then was filtered to remove insoluble inorganic salts. The solution was evaporated to dryness under reduced pressure. It was chromatographed by using CH2Cl2/acetone (30:1) to afford red solid in a 60% yield. ^{1}H NMR (400 MHz, d6-DMSO) δ: 8.70 (d, J = 8.3, 2H), 8.22 (dd, 2H), 7.89 (d, J = 8.0, 2H), 7.77 (d, J = 8.3, 2H), 7.66 (dd, J = 7.1, 2H), 7.56 (d, J = 5.1, 2H), 7.59 - 7.32 (dd, 4H), 7.22 - 7.15(m, J = 24.18H), 6.83 (dd, 2H), 6.73 (dd, J = 8.4, 2H), 5.60 (d, J = 2.1, 2H), 5.45 (d, J = 8.4, 2H), 1.0 - 0.71 (s, 18H). ^{13}C NMR (100 MHz, d6-DMSO, δ):180.08,

158.22, 156.27, 153.02, 150.25, 141.01, 138.34, 135.18, 134.08, 131.86, 130.77, 129.12, 127.50, 124.70, 115.90, 60.418, 58.49, 31.46, 29.53, 26.21, 22.07, 19.34, 14.32. MS (MALDI-TOF -MS) [m/z]: 1277.634 (4-PF6)$^+$.

Synthesis of L4: A mixture of 2-ethoxyethanol and water (3:1, v/v) was added to a flask containing $IrCl_3 \cdot 3H_2O$ (1 mmol) and **L1** (2.5 mmol). The mixture was refluxed for 24 h. After cooling down, the red solid precipitate was filtered and washed with ethanol to give crude cyclometalated Ir(III) chloro-bridged dimer.

Synthesis of 2: The solution of cyclometalated Ir(III) chloro-bridged dimer **L4** (200 mg) and *2,2'*-bipyridine (50 mg) in CH2Cl2/CH3OH [30 mL, 2:1 (v/v)] was heated to reflux. After 4 h, the red solution was cooled down to room temperature and then a 10-fold excess of potassium hexafluorophosphate was added. The suspension was stirred for 2 h and then was filtered to remove insoluble inorganic salts. The solution was evaporated to dryness under reduced pressure. It was chromatographed by using CH2Cl2/acetone (20:1) to afford red solid in a 60% yield. ^{1}H NMR (400 MHz, d6-DMSO) δ: 9.85 (s, 2H), 8.80 (d, J = 8.2 Hz, 1H), 8.33 - 8.24 (m, 1H), 8.11 m, J = 6.1 Hz, 2H), 7.84 (d, J = 8.4 Hz, 1H), 7.82 - 7.74 (m, 1H), 7.30 (m, J = 7.7 Hz, 1H), 7.07 (m, J = 7.8 Hz, 1H), 6.53 (dd, J = 8.4, 2.2 Hz, 1H), 5.94 (d, J = 8.4 Hz, 1H), 5.74 (s, 2H).

The CPEs and its procedure were synthesized according to the route depicted in Fig. S2.

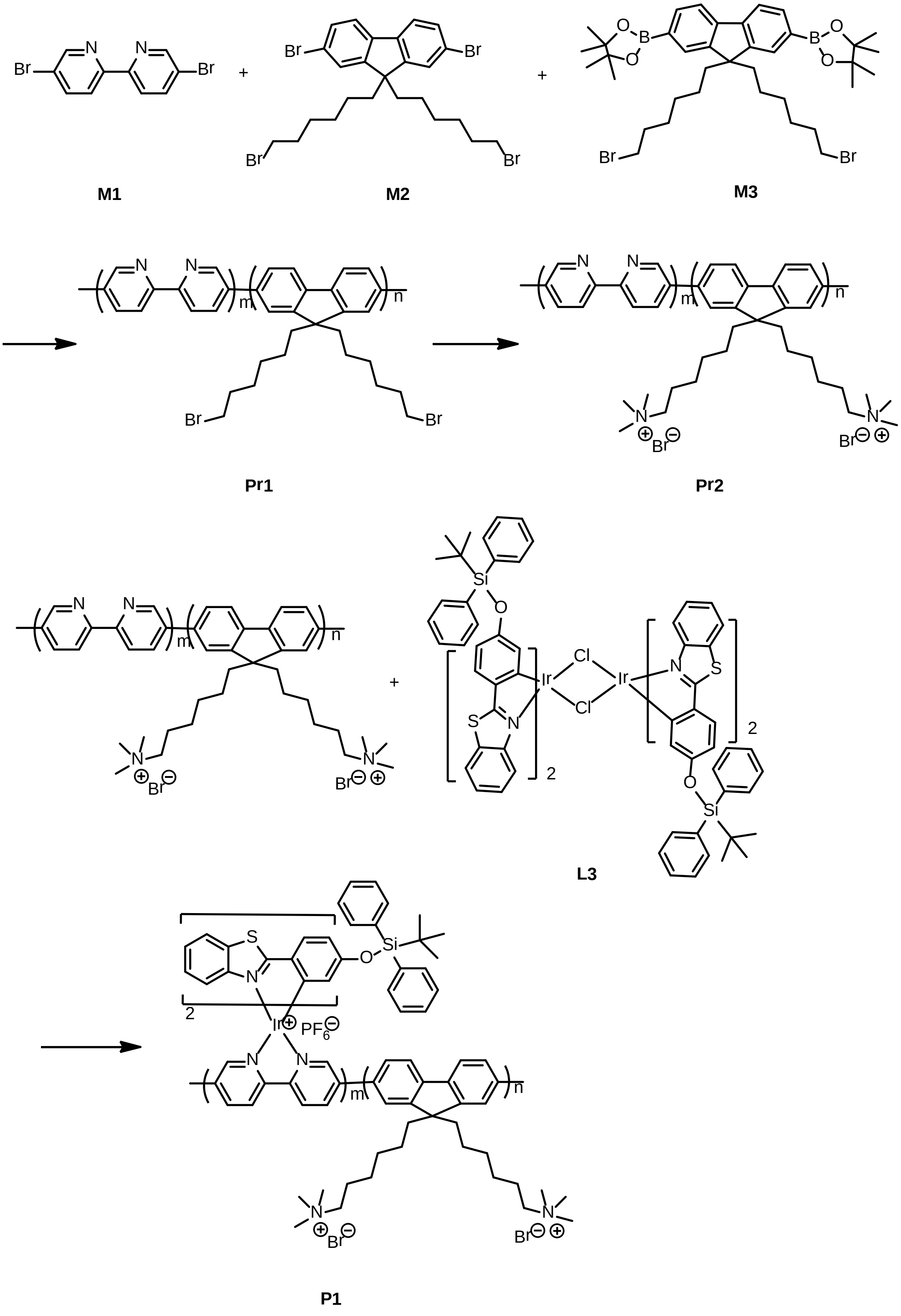

Figure S2. The synthetic routes of the CPEs.

Synthesis of **M1**, **M2** and **M3**: The monomers **M1**, **M2** and **M3** were synthesized according to the previous reports.[1-3]

The detail of synthetic of **P1** was stated in the methods part of the article.

2. Absorption spectra

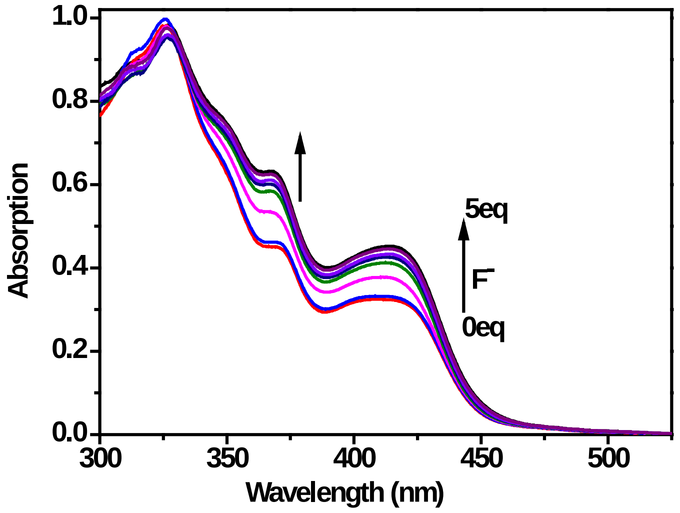

Figure S3. The response of absorption spectra of complex 1 to different concentrations of F- (with the addition of F- from 0 to 5-fold equivalents). The concentration of complex **1** was 1.26 × 10-4 mol/L. The test was carried out in tetrahydrofuran.

3. Mass spectra

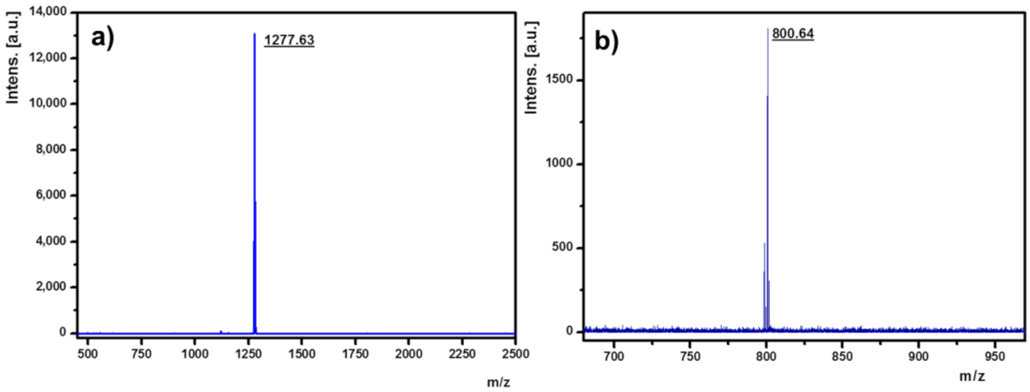

Figure S4. The mass spectra of compound **1** before (a) and after (b) addition of F^-.

4. Time-dependent PL spectra

The time-dependent PL spectrum of compound 1 with saturation concentrations of F^- was shown in Fig. S5. It can be seen that there is almost no time-dependent effects in the PL sensing process, and the emission intensity reaches to its saturation value within 60 seconds due to the rapid deprotection reaction.

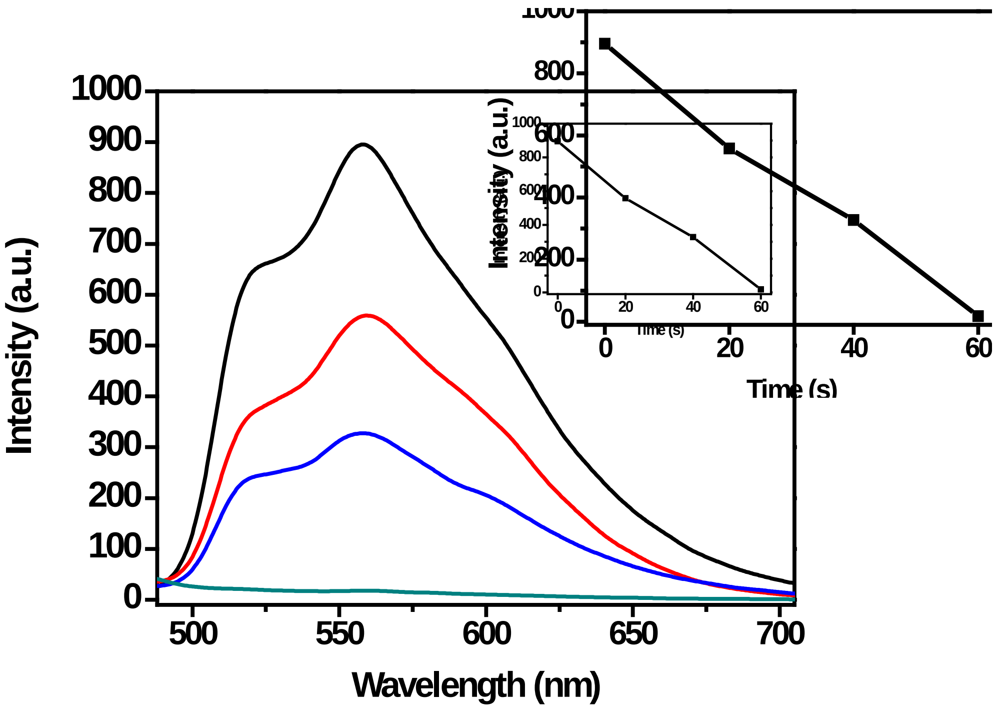

Figure S5. The time-dependent PL spectrum of compound 1 with saturation concentrations of F^-.

5. Ion selective response

Upon screening of a large variety of common anions, such as CO_3^{2-}, HSO_4^-, NO_2^-, NO_3^-, CH_3COO^-, HSO_3^-, HCO_3^-, Cl^-, Br^-, and F^-, probe **1** only exhibited significantly decreased emission (at 565 nm) in the presence of F^-. Other anions did not trigger any significant enhancement or disturb the response toward F^-.

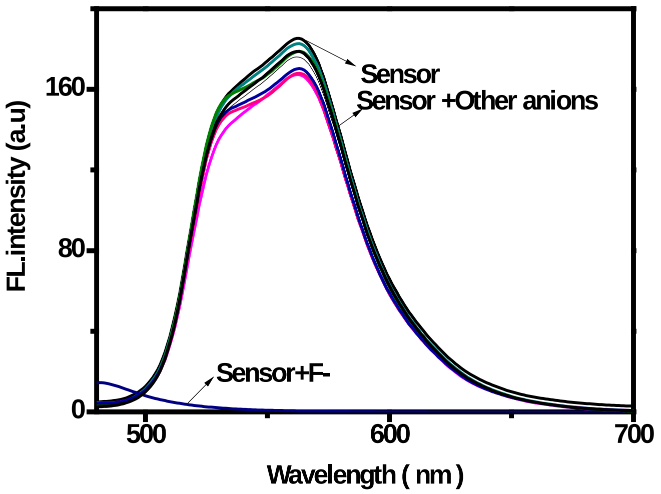

Figure S6. PL spectra (λ_{ex} = 370 nm) of complex **1** (20 μM) in the presence of 40 equivalents of various anions (CO_3^{2-}, HSO_4^-, NO_2^-, NO_3^-, CH_3COO^-, HSO_3^-, HCO_3^-, Cl^-, Br^-, and F^-) in THF.

6. Confocal images

To demonstrate the potential of the probe **1** in cellular sensing and imaging, the confocal images of living KB cells after incubation with probe **1** in PBS buffer for 60 min at 37 ℃. Then the experimental group was further incubated with F^- (20 μM) for 30 min at 37 ℃, and the control group was directly incubated for 90 min. We used confocal luminescence imaging to demonstrate the feasibility of the probe for F^- detection. The emission collected at 500-600 nm (B and E). As shown in the Fig. S5, after incubated with F^-, the lumienscence was significantly decreased, indicating that the probe exhibits good performances for F^- sensing and imaging.

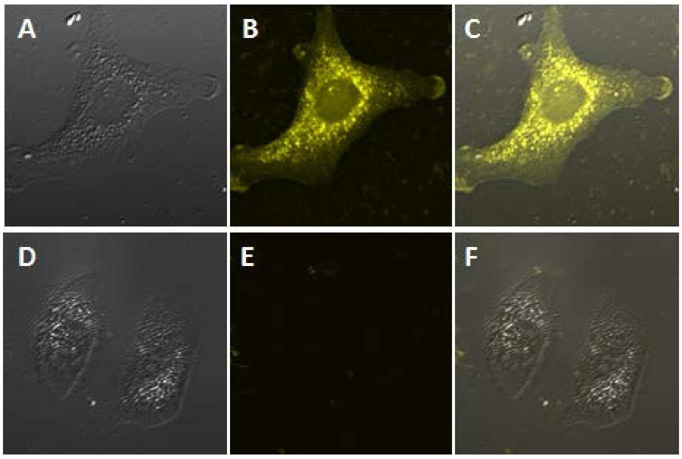

Figure S7. Confocal images of KB cell in the presence of the probe before (A-C) and after (D-F) incubated with F^-. Excitation wavelength was 405 nm; The A and D were bright-field, B and E was collected at 500-600 nm, and C and F were the overlay images. All the images share the same bar of 20 μm.

7. **Sensing performance of 1 and P1 under different pH values**

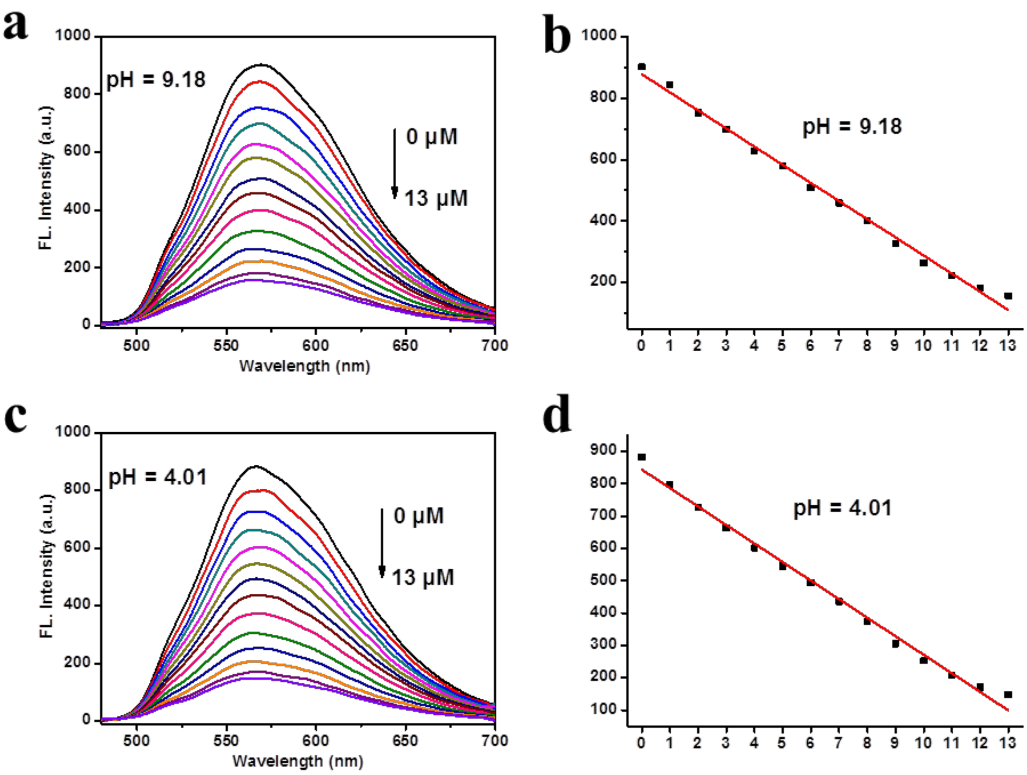

Figure S8. (a) PL spectra of complex **1** in sodium tetraborate solution (ST, pH=9.18) with addition of F^- from 0 to 13 μM upon excitation at 365 nm. (b) I_{600}/I_{420} as a function of [F^-] and its trendline in ST buffer solution. (c) PL spectra of complex **1** in potassium hydrogen phthalate solution (PHP, pH=4.00) with addition of F^- from 0 to 13 μM upon excitation at 365 nm. (d) I_{600}/I_{420} as a function of [F^-] and its trendline in PHP buffer solution.

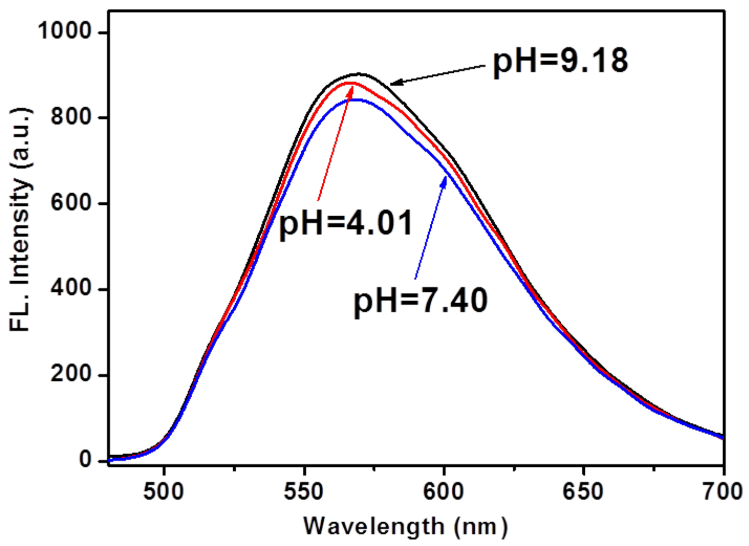

Figure S9. PL spectra of complex **1** in different pH buffer soulution without the addition of F^-.

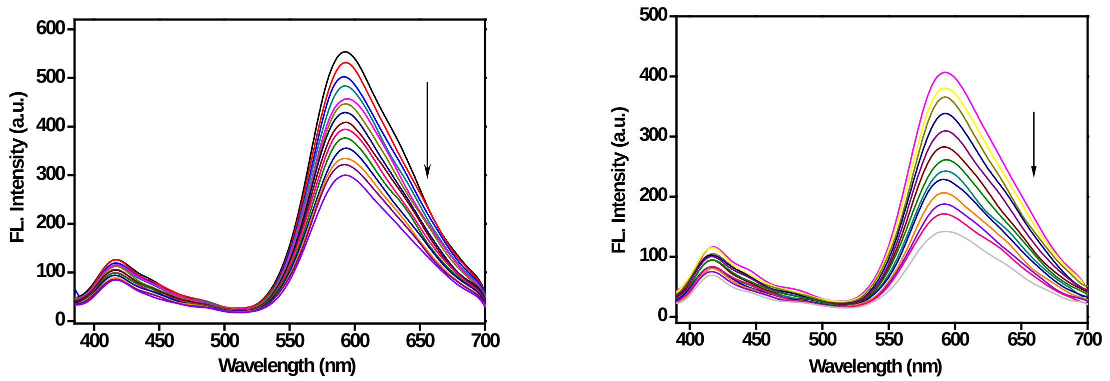

Figure S10. PL spectra of **P1** in aqueous solution under pH = 4.01 (left) or 9.18 (right) with addition of F^- from 0 to 13 μM upon excitation at 365 nm.

8. ^{1}H NMR

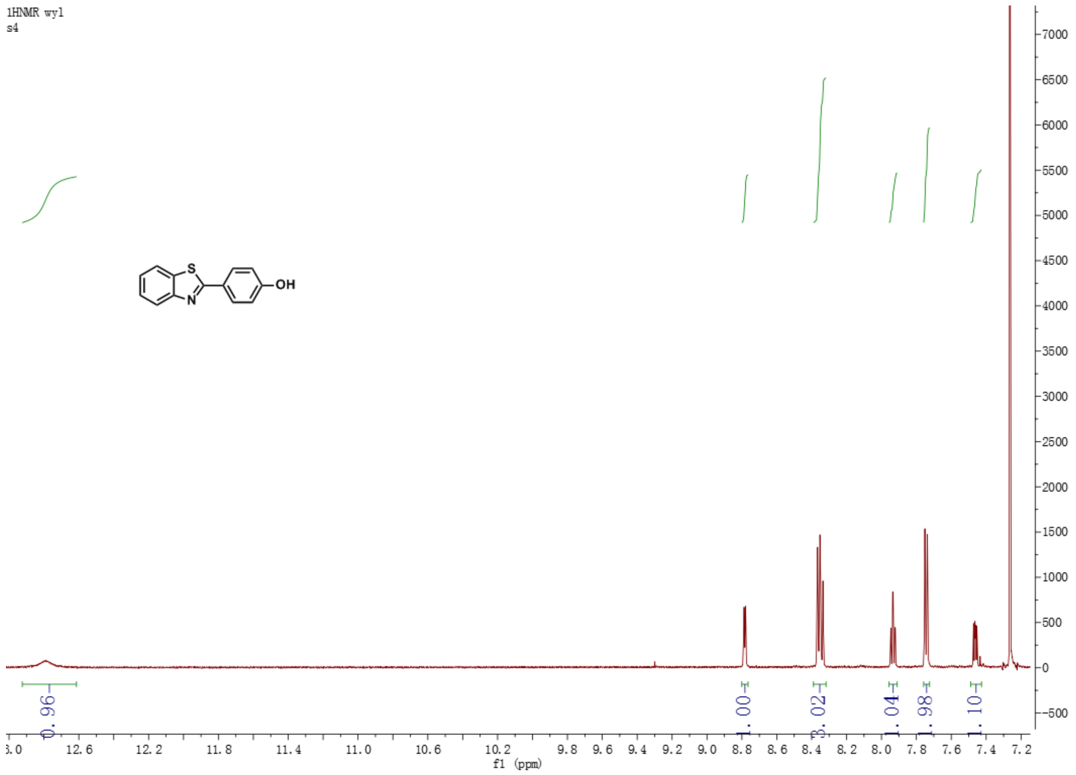
1HNMR wyl
s4
OH
S
N
0.96
1.00
3.02
1.04
1.98
1.10
f1 (ppm)

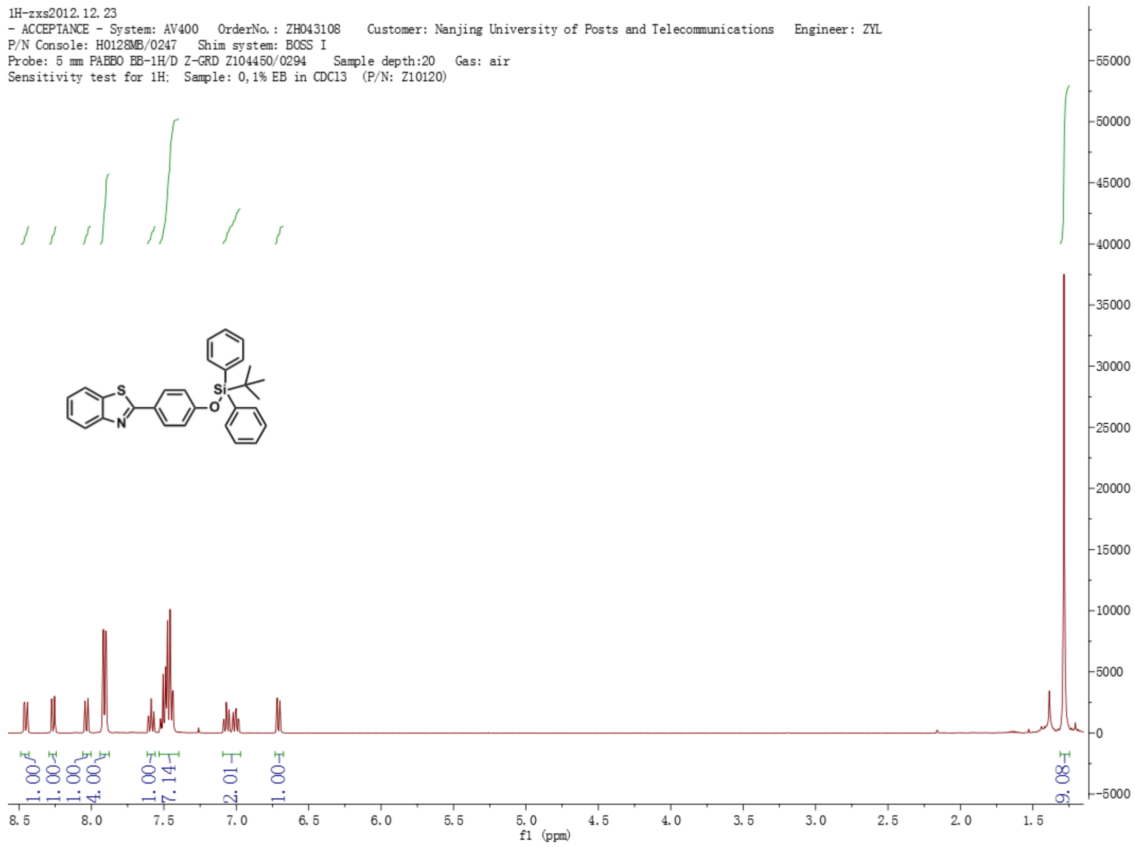
1H-zxs2012.12.23
- ACCEPTANCE - System: AV400 OrderNo.: ZH043108 Customer: Nanjing University of Posts and Telecommunications Engineer: ZYL
P/N Console: H0128MB/0247 Shim system: BOSS I
Probe: 5 mm PABBO BB-1H/D Z-GRD Z104450/0294 Sample depth:20 Gas: air
Sensitivity test for 1H; Sample: 0,1% EB in CDCl3 (P/N: Z10120)
S
N
O
Si
1.00
1.00
1.00
4.00
1.00
7.14
2.01
1.00
9.08
f1 (ppm)

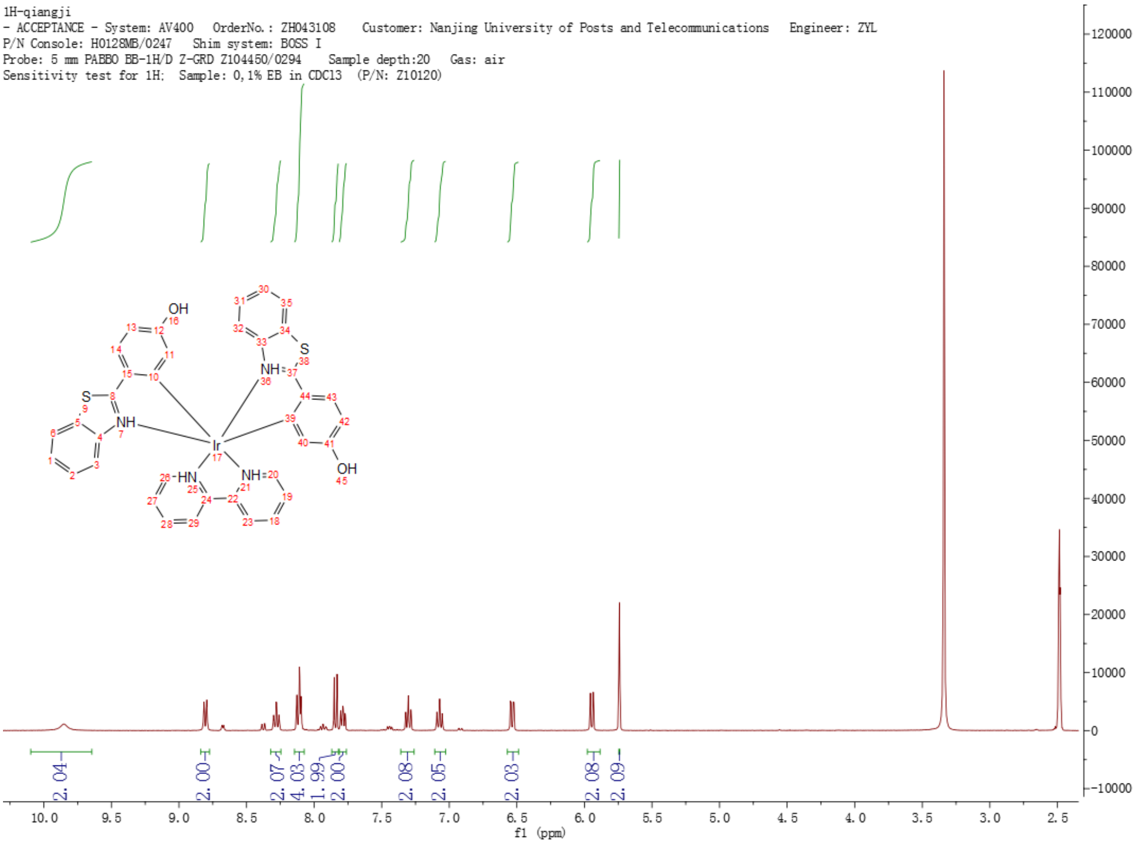
1H-qiangji
- ACCEPTANCE - System: AV400 OrderNo.: ZH043108 Customer: Nanjing University of Posts and Telecommunications Engineer: ZYL
P/N Console: H0128MB/0247 Shim system: BOSS I
Probe: 5 mm PABBO BB-1H/D Z-GRD Z104450/0294 Sample depth:20 Gas: air
Sensitivity test for 1H: Sample: 0,1% EB in CDCl3 (P/N: Z10120)
f1 (ppm)

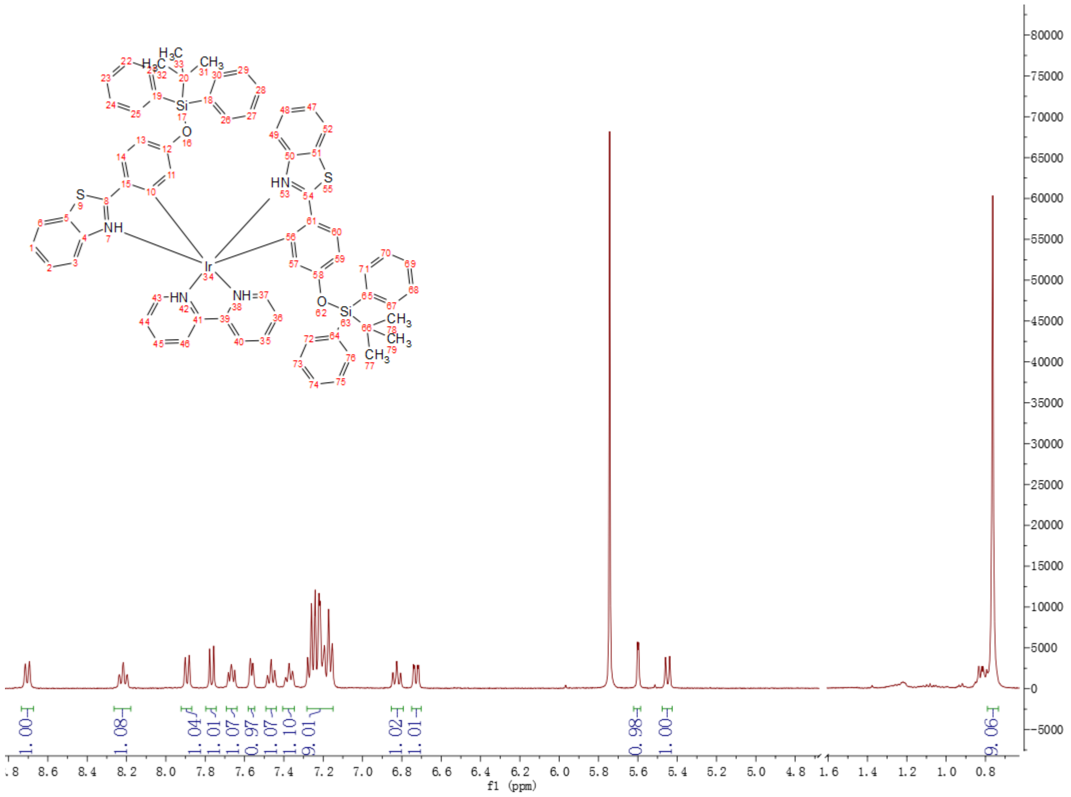
f1 (ppm)

9. References

1. Newkome,G. R., Patri, A. K., Holder, E. & Schubert, U. S. Synthesis of 2, 2 '-bipyridines: Versatile building blocks for sexy architectures and functional nanomaterials. *Eur. J. Org. Chem.* **2**, 235-254 (2004).
2. Shi, H. F. et al. Simple Conjugated Polymers with On-Chain Phosphorescent Iridium(III) Complexes: Toward Ratiometric Chemodosimeters for Detecting Trace Amounts of Mercury(II). *Chem. Eur. J.* **16**,12158-12167 (2010).
3. Liu, B. & Bazan, G. C. Synthesis of cationic conjugated polymers for use in label-free DNA microarrays. *Nat. Proctoc.* **1**, 1698-1702 (2006).
